# Gene expression profiling in nucleus pulposus of human ruptured lumbar disc herniation

**DOI:** 10.3389/fphar.2022.892594

**Published:** 2022-11-25

**Authors:** Xiaochun Li, Xueqiang Shen, Zhiqiang Wang, Hong Jiang, Zhijia Ma, Pengfei Yu, Zhenhan Yu, Xiang Qian, Jintao Liu

**Affiliations:** ^1^ Suzhou Hospital of Traditional Chinese Medicine, Suzhou, China; ^2^ Nanjing University of Traditional Chinese Medicine, Nanjing, China

**Keywords:** ruptured, lumbar disc herniation, nucleus pulposus, gene chip, gene

## Abstract

**Purpose:** To examine the differences in gene expression between ruptured and non-ruptured nucleus pulposus tissues of the intervertebral discs using gene chip technology.

**Methods:** A total of 8 patients with nucleus pulposus from a lumbar disc herniation (LDH) undergoing discectomy in our hospital were selected, including 4 ruptured and 4 non-ruptured herniated nucleus pulposus cases. Total RNA was extracted from cells by using TRIzol reagent. Nucleus pulposus cDNA probes of the two groups were obtained by the single marker method and hybridized with a human gene expression profiling chip (Agilent). The fluorescence signal images were scanned by a laser, and the obtained genes were analyzed by bioinformatics.

**Results:** There were 75 differentially expressed genes with more than 2-fold-changes, of which 56 were up-regulated and 19 were down-regulated. The differential expression of THSD7A, which was up-regulated 18 times, was the most significant, followed by CCL5, AQP3 and SDC4.

**Conclusion:** THSD7A can be used as a characteristic differentially expressed gene in human ruptured nucleus pulposus. Moreover, CCL5, AQP3 and SDC4 may improve the chemotaxis of stem cell migration for self-healing of damaged disc tissue, increase water uptake by nucleus accumbens cells, and inhibit the inflammatory response, thus delaying the process of intervertebral disc degeneration.

## Introduction

Intervertebral disc degeneration is a medical condition characterized by degeneration of the discs that separate the bone from the spine (Kadow et al., 2015). Increased intervertebral disc pressure, insufficient nutritional supply, inflammatory response, and genetic factors are the major causes of this condition (Le Maitre et al., 2009); yet, its exact pathogenesis is still unknown.

Lumbar disc herniation (LDH) is the most common intervertebral disc degeneration caused by annular fiber disruption with a discrete leakage of nucleus pulposus pressing on a nerve (Kepler et al., 2013b). It can be divided into ruptured and non-ruptured types (Jiang et al., 1998; Li et al., 2010). Ruptured LDH is the rupture of the annulus fibrosus of the disc, herniation of the nucleus pulposus, and herniation through the posterior longitudinal ligament. The non-ruptured type is usually referred to as the nonrupture of the annulus fibrosus. Disk degeneration of the ruptured type is usually more severe and more prone to reabsorption after herniation (Gao et al., 2017; Yu et al., 2018). Degeneration can disrupt the homeostasis of the extracellular matrix of the intervertebral disc. The balance of anabolism and catabolism in the normal intervertebral disc extracellular matrix is mainly maintained by a disintegrin and metalloproteinase with thrombospondin-like motifs (ADAMTSs), matrix metalloproteinases (MMPs), and tissue inhibitor of metalloproteinasas (TIMPs). When intervertebral disc degeneration occurs, an imbalance in the expression of cytokines leads to reduced matrix synthesis and increased production of matrix-degrading enzymes, resulting in accelerated degradation and loss of the hydration status of the extracellular matrix, which in turn accelerate the degeneration process (Yasuma et al., 1990). However, the ruptured type may initiate self-repair and delay degeneration through the posterior longitudinal ligament after herniation. Nonetheless, the exact mechanism still remains unclear.

Gene chip, also known as DNA microarray, is the most widely used and most mature technology. It works on a similar principle to Southern and Northern blot hybridization. Yet, it is several thousand-fold more powerful than the northern blot for studying gene expression. This technology has great value in large-scale gene discovery, gene analysis, genome research, and especially in gene expression profiling (De Benedetti et al., 2000).

Currently, the use of gene chip technology to study intervertebral disc degeneration diseases has become a new direction in scientific research. In this study, we evaluated the differences in gene expression between ruptured and non-ruptured nucleus pulposus tissues of the intervertebral disc using gene chip technology.

## Materials and methods

### Reagents and equipment

Thermal cycler (MJ, US), Hybridization Oven, Hybridization Kit, Chip scanner (Agilent Technologies Santa Clara, US), Microuv/visible spectrophotometer (Nanodrop, US); RNeasy mini Kit (250), RNeasy micro Kit (50), RNase-free DNase set (50) (QIAGEN GmBH, Germany), Low Input Quick Amp Labeling Kit RNA Spike-In Kit, One Color, Gene Expression Hybridization Kit, Gene Expression Wash Buller Kit (Agilent Technologies Santa Clara, US) were used in this study.

### Specimen collection

Eight nucleus pulposus tissues from LDH patients undergoing discectomy in our hospital were collected. The specimens collected intraoperatively were immediately fixed into RNA-late solution and stored at low temperature after the fluid was infiltrated into the tissues. Patients were divided into ruptured group and non-ruptured group according to the intactness of the posterior longitudinal ligament revealed by magnetic resonance imaging (MRI). There were 4 patients with ruptured nucleus pulposus and four patients with nonuptured nucleus pulposus (4 males and 4 females), aged 35–71 years (average age of 54.8 years old). The studies involving human participants were reviewed and approved by the Ethics Committee of Suzhou Hospital of Traditional Chinese Medicine. The patients provided written informed consent to participate in this study.

## Detection by gene chip

### Total RNA extraction

Preparation of 75% alcohol: A total of 37.5 ml anhydrous ethanol was mixed with 12.5 ml nuclease-free water. The centrifugal speed in all steps was 13,200 rpm. Buffer PRE was prepared following the manufacturer’s instructions. The centrifugal machine was pre-cooled at 4°C. The intervertebral disc tissues were then grounded in the liquid nitrogen by a high-throughput tissue grinder and homogenized for approximately 5 min. Then, 1 ml of TRIzol was vortexed with 1/5 volume of chloroform for about 15 s, then left at room temperature for 5 min, and centrifuged under 13,200 rpm for 15 min at 4°C. The supernatant was carefully removed with a 200 μL pipette, avoiding touching the middle layer, transferred into a new 1.5 ml centrifuge tube, and gently mixed upside down with 1.5 times the volume of anhydrous ethanol.

Consequently, 700 μL solution containing total RNA was transferred into an RNeasy mini centrifuge column in a 2 ml collection tube, centrifuged at least 10,000 rpm for 15 s, after which the filtrate was discarded. The last step was repeated until the mixed solution was fully-added to the RNeasy mini centrifugal column. Then, 350 μL Buffer RW1 was added and centrifuged at 10,000 rpm for 15 s, after which the filtrate was discarded. The prepared mixed solution of 70 μL RDD and 10 μL DNaseI was then added to the purified column and let stand at room temperature for 15 min. Next, 350 μL Buffer RW1 was added to the solution and centrifuged at 10,000 rpm for 15 s, after which the filtrate was discarded. Then, 500 μL Buffer RPE was added in the RNeasy mini centrifugal column and centrifuged for rinsing at least 10,000 rpm for 15 s, after which the filtrate was discarded. Consequently, 500 μL Buffer RPE was used for rinsing and centrifuged at least 10,000 rpm for 2 min. The filtration liquid was discarded, and the 2 ml collection tube was removed.

The RNeasy mini centrifugal column was transferred into a new tube. After gently closing the lid, the tubes were centrifuged at full speed for 1 min. The RNeasy centrifugal column was placed in a new 1.5 ml collection tube. Next, 30–50 μL RNase-free water was added directly to the RNeasy mini membrane. After closing the lid, the samples were let stand at room temperature for 1 min and centrifuged at least 10,000 rpm for 1 min. The eluent was re-added to the RNeasy mini column membrane and let stand at room temperature for 1 min. Then, the RNA was eluted by centrifugation at least 10,000 rpm for 1 min. The obtained samples were stored at −80°C.

### Purification of total RNA

Total RNA100 µg was dissolved in 100 µL RNase-free water and thoroughly mixed with 350 µL Buffer RLT, after which 250 µL anhydrous ethanol was added and thoroughly mixed with the tip. A total of 700 µL solution containing total RNA was transferred into the RNeasy column in a 2 ml centrifuge tube, centrifuged at 13,200 rpm for 15 s, after which the filtrate was discarded. Next, 350 µL Buffer RW1 was transferred into the RNeasy mini-column, centrifuged at 13,200 rpm for 15 s, and the filtrate was discarded. Then, 10 µL DNase I was mixed with 70 µL Buffer RDD, after which 80 µL of the mixed solution was added to the column and let stand at room temperature for 15 min. Next, 350 µL Buffer RW1 was added into the RNeasy mini-column, centrifuged at 13,200 rpm for 15 s, and the filtrate was discarded. Consequently, 500 µL Buffer RPE was added to the RNeasy mini-column, centrifuged at 13,200 rpm for 15 s, after which the filtration liquid was discarded. The last step was repeated once again. A new collection tube was replaced, followed by centrifugation at 13,200 rpm for 2 min, and the column was transferred into an elution tube. The RNeasy mini-column was transferred into the collection tube. Then, 30 µL RNase-free water was left for 1 min, centrifuged at 13,200 rpm for 1 min, after which 30 μL of the sample in the elution tube was transferred back to the column, let stand for 1 min, and centrifuged at 13,200 rpm for 1 min. A NanoDrop was used to measure the RNA concentration at 260/280 nm.

### Reverse transcription

According to the initial amount of RNA, a spike-in of a single mark was prepared. A reaction solution was also prepared. The temperature of 65°C was kept in the PCR device for 10 min and the ice bath for 5 min. At the same time, 5X first-strand buffer was preheated at 80°C for 3 min in standby at room temperature. A reverse transcription mix was then prepared. A tota of 4.7 µL of the above mix was added to the denatured RNA in the ice bath, mixed well, and centrifuged. PCR reaction was performed as follows: 40°C for 2 h; inactivation at 70°C for 15 min; 4°C for 5 min.

### Fluorescence labeling and product purification

The labeled mix was prepared. Then, 6.0 µL mix was added for mixing, followed by centrifugation. PCR reaction was performed as follows: 40°C for 2 h; 4°C for 5 min. Next, 84 µL nuclease-free water was added to make the volume 100 μL; 350 µL RLT was added for mixing, and 250 µL anhydrous ethanol was added for mixing without centrifugation. Consequently, 700 µL mix was transferred to the column centrifuged under 13,000 rpm at 4°C for 30 s, after which the eluent was discarded. Next, 500 μL RPE was added and centrifuged under 13,000 rpm at 4°C for 30 s. The eluent was again discarded. Another 500 μL RPE was then added to the solution and centrifuged under 13,000 rpm at 4°C for 60 s, after which the eluent was discarded. A new tube was used for replacement and centrifuged under 13,000 rpm without loading anything at 4°C for 30 s. The column was transferred into the eluting tube and mixed with 30 µL nuclease-free water. The column was let stand for 1 min and centrifuged under 13,000 rpm at 4°C for 30 s. The 30 µL sample in the elution tube was transferred back to the column and let stand for 1 min at 13,000 rpm, centrifuged at 4°C for 30 s. RNA and Cy3 concentrations were measured at 260/280 with a NanoDrop.

### Chip hybridization

The fragmented mix was prepared, heated at 60°C for 30 min, cooled in the ice bath for 1 min, and centrifuged for a short time. The 2X GEx hybridized Buffer HI-rpm with equal volume was added for mixing, centrifuged at 13,000 rpm for 1 min, and put on ice. The hybridization chamber was placed on a horizontal tabletop with a cover glass with a washer. Samples were then added. The chip with “Agilent” was covered face down on the cover glass. The hybridization chamber was quickly assembled and placed in a hybridization oven at 65°C and 10 rpm for 17 h.

### Chip rinsing and scanning

A 2 ml 10% Triton X-102 was added to washing solutions 1 and 2; the washing solution 2 was preheated overnight at 37°C. Hybridization chips were taken out from the oven, and the chamber was opened. The chip was then rinsed following the instructions. The rinsed chips were loaded into the clip and placed into the scanner for scanning.

## Bioinformatics analysis

### Screening for differential expression genes

The raw data were normalized by limma package in the R software. Fold-change and Student’s *t*-test were used to screen the differentially expressed genes, as follows: Fold Change (Linear) =< 0.5 or ≥ 2; *t*-test *p*-value < 0.05.

### Scatter plot, volcano plot, and heatmap

The raw data of the chips were standardized and converted into log base 2. Then, the scatter plot was drawn in a two-dimensional rectangular coordinate system plane. The scatter plot is often used to evaluate the central tendency of the overall distribution of the two data groups. Each point in the plot represents a probe point on the chip, and the position of the point in the two-dimensional plane is determined by the X-axis (normalized signal value of the point in the control chip) and Y-axis (normalized signal value of the point in the sample chip). The *p*-value and fold change obtained by *t*-test between Agilent chip groups were used to draw a volcano plot to indicate the significant difference between the two sample groups. In the volcano plot, one of the coordinates showed the negative log of *p*-values calculated by *t*-test, and the other showed the value change of log2 compared under two conditions. Heatmap is an intuitive graphic representation of gene expression in different samples after hierarchical clustering of samples and genes.

### GO enrichment analysis and KEGG enrichment analysis of differential genes

The upregulated and downregulated differential genes were imported into the GO database and KEGG PATHWAY database for analysis, respectively. *p* < 0.05 was considered a statistically significant difference. GO analysis can be used for enrichment analysis from molecular function, biological process, and cellular component. KEGG enrichment analysis can enrich the pathways with significant differences and help to identify biological regulatory pathways with significantly different changes under experimental conditions. The two methods are similar. GO analysis is Fisher’s Exact Test, with the data packets from clusterProfiles of R/bioconductor. The selection criteria were the number of differentially expressed genes on a term/GO ≥ 2, *p*-value < 0.05. Term/GO was obtained by taking the top 30 results in descending order from the values of enriching factor (number of different genes in a term/total number of different genes in the database)/(total number of genes in a term in the database/total number of genes in the database). The KEGG pathways with enrichment functions were obtained *via* the GO enrichment principle.

### Chip verification

Five genes were randomly selected according to the gene expression profiling in the nucleus pulposus. Primer Express 3.0.1 software was used to design and synthesize the gene primers to be verified, and the first strand of cDNA was synthesized. Dissolution curves were plotted and analysed using SYBR Green qPCR.

## Results

### Differential expression genes

**FIGURE 1:**
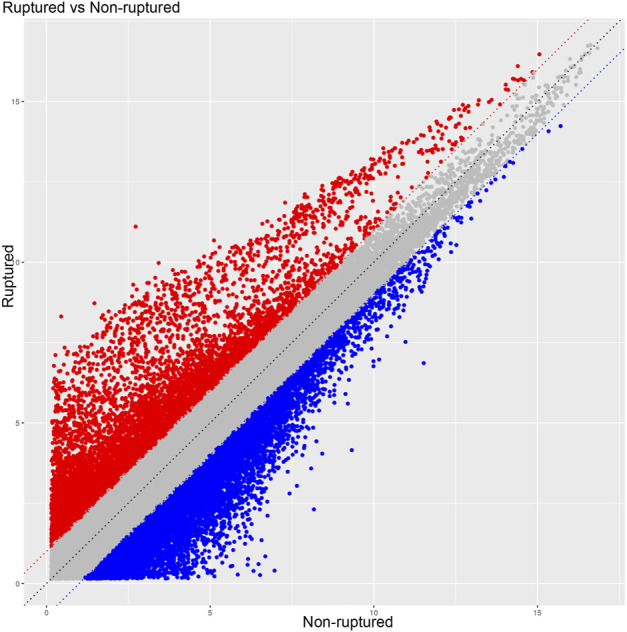
Scatter plot.

**FIGURE 2:**
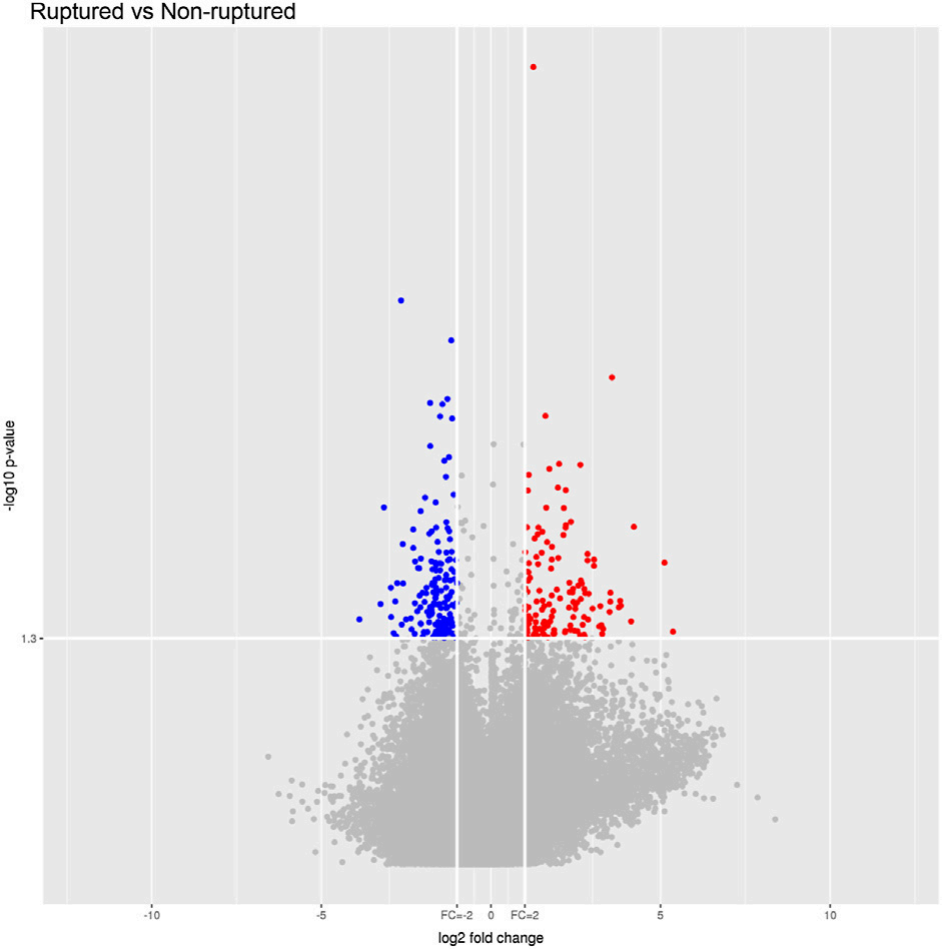
Volcano plot.

**FIGURE 3:**
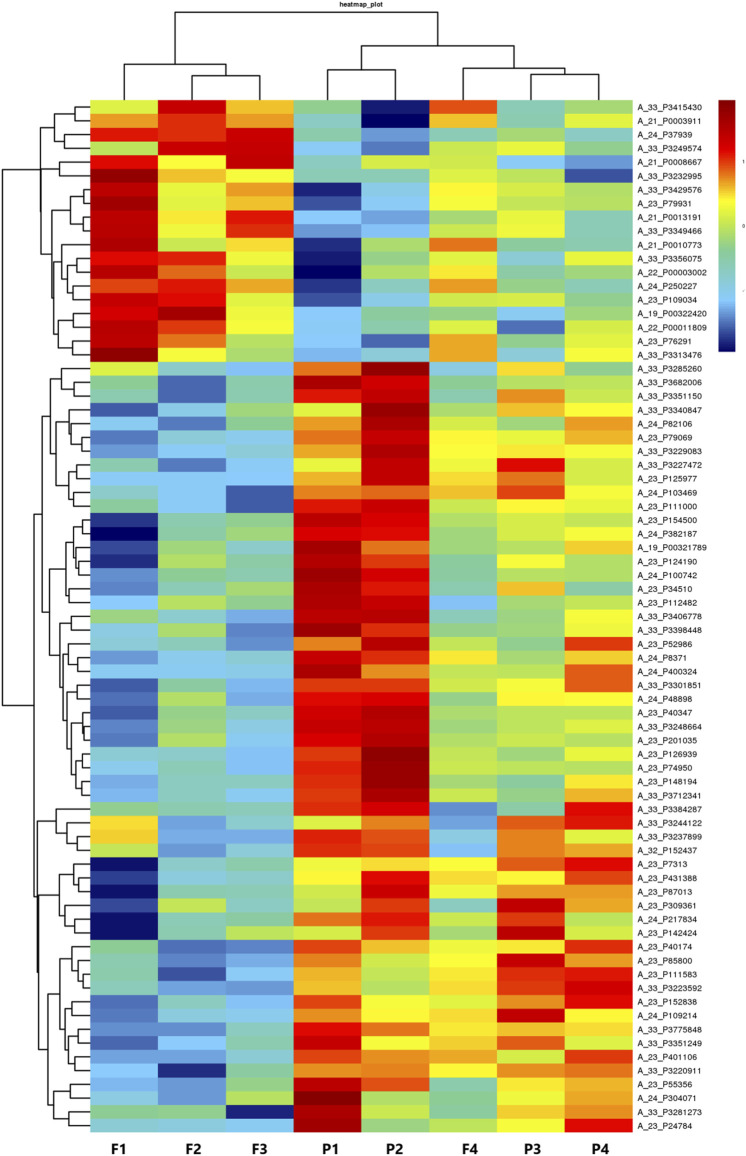
Heatmap.

After the raw data were normalized by the limma package in the R software, fold-change and Student’s *t*-test were used to screen the differentially expressed genes, as follows: Fold Change (Linear) =< 0.5 or ≥ 2; *t*-test *p*-value < 0.05. The following selection conditions were applied: Fold Change (linear) ≤0.5 or Fold Change (linear) ≥2; *t*-test *p*-value < 0.05. There were 75 differentially expressed genes with more than 2 fold changes, of which 56 genes were up-regulated and 19 genes were down-regulated. Scatter plot (Figure 1), volcano plot (Figure 2), and heatmap (Figure 3) were then drawn based on the differentially expressed genes. The expression of THSD7A, which was up-regulated 18 times, was the most significant, followed by CCL5, AQP3, and SDC4 expression (Tables 1, 2).

**TABLE 1:** Differentially expressed genes were up-regulated (Fold change>2).

Genbank	GeneSymbol	Foldchange	Description
NM_003530	HIST1H3D	2.15439714340075	histone cluster 1, H3d
NM_175629	DNMT3A	3.56754647448082	DNA (cytosine-5-)-methyltransferase 3 alpha
NM_012205	HAAO	2.02866203843658	3-hydroxyanthranilate 3,4-dioxygenase
NM_002985	CCL5	8.22170604583032	chemokine (C-C motif) ligand 5
NM_138432	SDSL	6.87033352448876	serine dehydratase-like
NM_018269	ADI1	2.71072412204743	acireductone dioxygenase 1
NM_032587	CARD6	4.97154757755622	caspase recruitment domain family, member 6
NM_003929	RAB29	5.06959463692048	RAB29, member RAS oncogene family
NM_001645	APOC1	14.2146534986589	apolipoprotein C-I
NM_144569	SPOCD1	6.51835128637505	SPOC domain containing 1
NM_001289	CLIC2	4.93093174601446	chloride intracellular channel 2
NM_001001522	TAGLN	6.71207538407453	transgelin
NM_002419	MAP3K11	2.52832177925584	mitogen-activated protein kinase kinase 11
NM_152718	VWCE	3.84259885720114	von Willebrand factor C and EGF domains
NM_001293070	CHN2	5.61330393294964	chimerin 2
NM_001124758	SPNS2	6.70813077161383	spinster homolog 2 (Drosophila)
NM_182566	VMO1	3.04377071465793	vitelline membrane outer layer 1 homolog (chicken)
NM_006295	VARS	2.01430531159549	valyl-tRNA synthetase
NM_198040	PHC2	3.63656846069221	polyhomeotic homolog 2 (Drosophila)
NM_130390	TRIM34	5.38525722602181	tripartite motif containing 34
NM_001001547	CD36	9.90186086477477	CD36 molecule (thrombospondin receptor)
NM_004925	AQP3	9.71774027599458	aquaporin 3 (Gill blood group)
NM_001040058	SPP1	6.192357681748	secreted phosphoprotein 1
NM_172369	C1QC	11.4852446679992	complement component 1, q subcomponent, C chain
NM_001100812	CXCL16	3.58155329413271	chemokine (C-X-C motif) ligand 16
NM_001033886	CXCL12	5.35494787430273	chemokine (C-X-C motif) ligand 12
NM_178580	HM13	2.97647116446788	histocompatibility (minor) 13
NM_002579	PALM	2.10845581900773	paralemmin
NM_014189	ADD1	2.97492160222571	adducin 1 (alpha)
NM_001005741	GBA	2.92423309128731	glucosidase, beta, acid
NR_102735	DBH-AS1	17.5301415078466	DBH antisense RNA 1
NM_002599	PDE2A	6.47267131941332	phosphodiesterase 2A, cGMP-stimulated
NM_005100	AKAP12	4.43407135664533	A kinase (PRKA) anchor protein 12
NM_022904	RASAL3	5.96824111794013	RAS protein activator like 3
NM_003775	S1PR4	6.27896394084608	sphingosine-1-phosphate receptor 4
NM_001547	IFIT2	5.08193565823766	interferon-induced protein with tetratricopeptide repeats 2
NM_007161	LST1	5.50231079453248	leukocyte specific transcript 1
NM_001552	IGFBP4	11.2953295271502	insulin-like growth factor-binding protein 4
NM_015204	THSD7A	18.5545930209694	thrombospondin, type I, domain containing 7A
NM_004995	MMP14	2.49618825851842	matrix metallopeptidase 14 (membrane-inserted)
NM_003282	TNNI2	7.97989829664715	troponin I type 2 (skeletal, fast)
NM_004994	MMP9	13.6486218452929	matrix metallopeptidase 9 (gelatinase B, 92 kDa gelatinase, 92 kDa type IV collagenase)
NM_032789	PARP10	3.36086917881329	poly (ADP-ribose) polymerase family, member 10
NM_145637	APOL2	2.59953965936267	apolipoprotein L, 2
NM_004335	BST2	6.82199688565174	bone marrow stromal cell antigen 2
NM_001302688	APOE	9.56125791305111	apolipoprotein E
NM_002800	PSMB9	2.82017393059116	proteasome (prosome, macropain) subunit, beta type, 9
NM_144584	HENMT1	2.01845494401461	HEN1 methyltransferase homolog 1 (Arabidopsis)
NM_001803	CD52	14.0161596478736	CD52 molecule
NM_080593	HIST1H2BK	2.2225251624331	histone cluster 1, H2bk
NM_022068	PIEZO2	6.21356113215153	piezo-type mechanosensitive ion channel component 2
NM_024660	IGFLR1	2.39177923541253	IGF-like family receptor 1
NM_018715	RCC2	2.10345473495254	regulator of chromosome condensation 2

**TABLE 2:** Differentially expressed genes were down-regulated (Fold change<0.5).

Genbank	GeneSymbol	Foldchange	Description
NM_007244	PRR4	0.410211377339053	proline rich 4 (lacrimal)
NM_021724	NR1D1	0.112333480199683	nuclear receptor subfamily 1, group D, member 1
NM_002999	SDC4	0.38691026053581	syndecan 4
NR_033856	FLJ43315	0.147218857926714	asparagine synthetase pseudogene
NM_001136571	ZAR1L	0.143924879643004	zygote arrest 1-like
NM_001261429	LPIN1	0.443379785749477	lipin 1
XR_424667	LOC102724566	0.204613784629522	uncharacterized LOC102724566
NM_001284	AP3S1	0.395163396964712	adaptor-related protein complex 3, sigma 1 subunit
NM_001204056	ANKRD12	0.266377248497569	ankyrin repeat domain 12
NM_139322	ATRN	0.194633025291779	attractin
NM_005346	HSPA1B	0.473298533927247	heat shock 70 kDa protein 1B
NM_004866	SCAMP1	0.429786214059183	secretory carrier membrane protein 1
NM_198795	TDRD1	0.308072001433343	tudor domain containing 1

### GO and KEGG enrichment analysis

**FIGURE 4:**
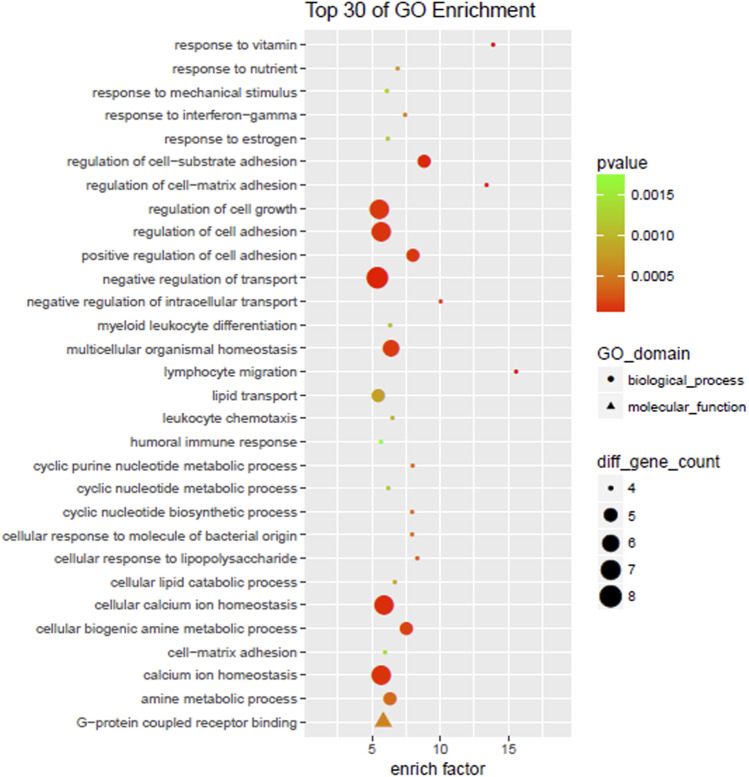
GO enrichment.

Most of the top 30 items were biological processes in all GO levels enriched by differentially expressed genes with fold change ≥ 2 and a *p*-value < 0.05, including lymphocyte migration, cell-matrix a dhesion, cyclic nucleotide biosynthesis, cell lipid catabolism, leukocyte chemotaxis, homeostasis in multicellular organisms, humoral immune response, cell growth regulation, G protein-coupled receptor binding, etc. (Figure 4).

**FIGURE 5:**
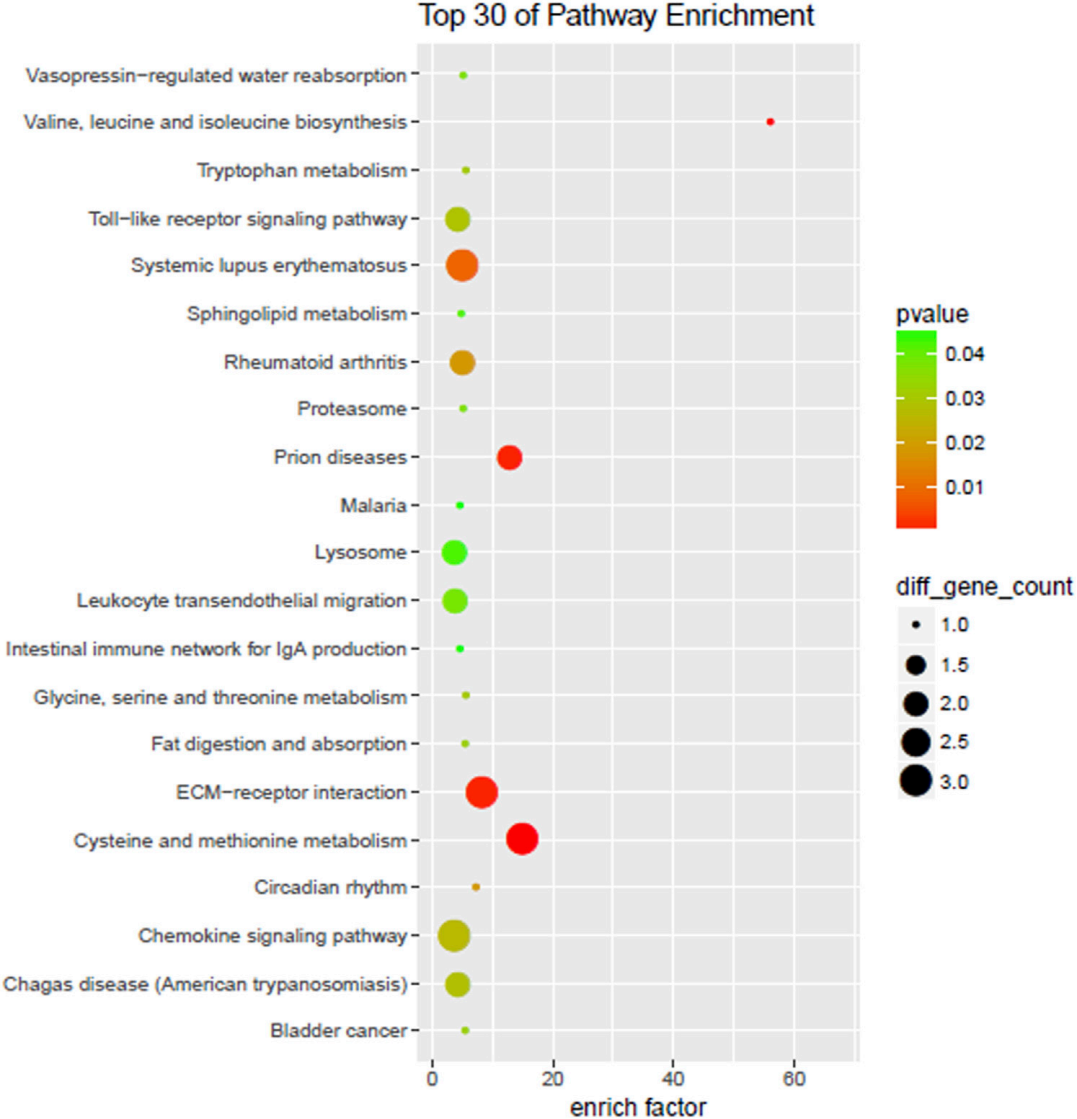
KEGG enrichment.

Differentially expressed genes enriched all KEGG with fold change ≥ 2 and a *p*-value < 0.05. The top 30 pathways included ECM-receptor interaction, vasopressin-regulated water reabsorption, Toll-like receptor signaling pathway, chemokine signaling pathway, amino acid synthesis, and metabolism (Figure 5).

### Chip verification

**FIGURE 6:**
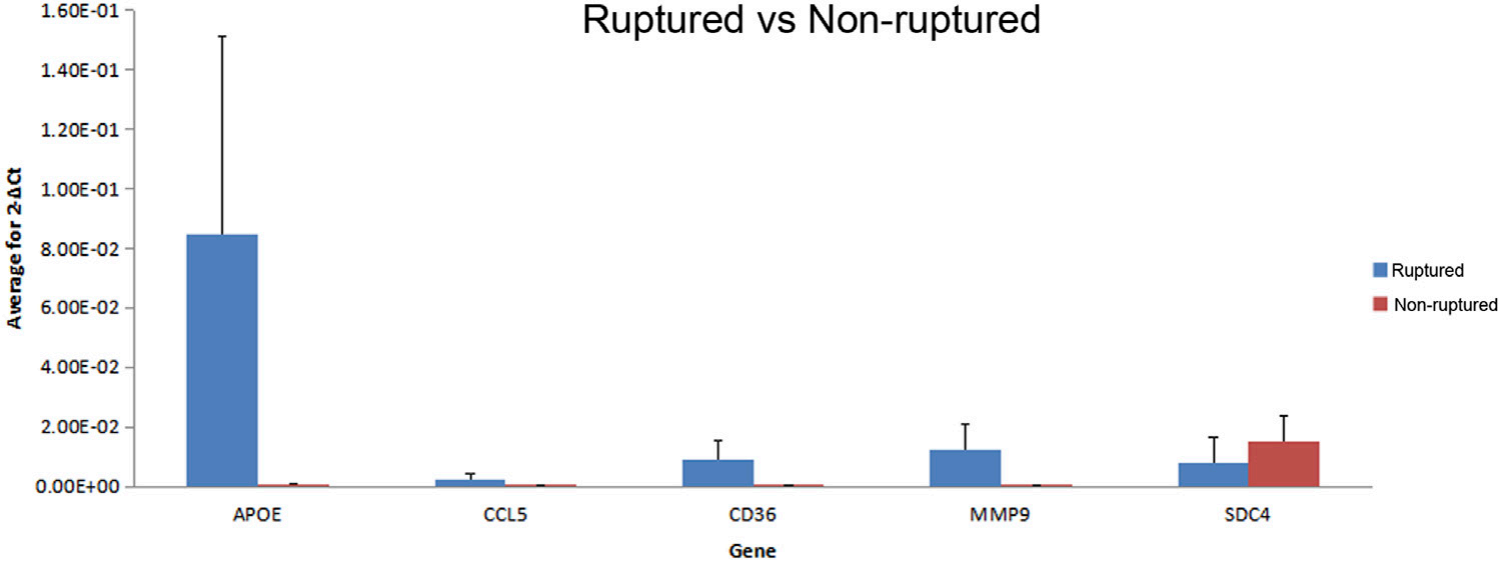
The verification results of randomly extracted differential expression gene.

All results showed a single peak *via* dissolution curve analysis, indicating good PCR amplification specificity. The repeatability in triplicated experiments was good, and the results were significantly different (*p* < 0.05), which showed the same trend as the chip results. All the sampled genes were successfully verified, which indicated that the chip results were relatively reliable (Figure 6).

## Discussion

In this study, we used a gene chip to examine the differences in gene expression between ruptured and non-ruptured nucleus pulposus tissues of the intervertebral disc. THSD7A, followed by CCL5, AQP3, and SDC4, were significantly different between the two groups.

Thrombospondin Type I Domain containing 7A (THSD7A) is a member of the KIAA family. The Thsd7a gene is located at G-B7p23.3 and has a length of 458 kb. The chromosome 7p21 sequence is common in the frequent and vulnerable regions of chromosomal abnormalities in various tumors (Bosco et al., 2010). Studies have shown that the THSD7A acts *via* the TGF-β (common in non-tumor diseases) or mTOR signaling pathways (common in tumor diseases) (Young and Murphy-Ullrich, 2004). Current studies on THSD7A-related diseases mainly include osteoporosis, membranous nephropathy, and tumors. Zhou et al. (2013) found that osteoporotic spinal fracture was closely associated with THSD7A and N substructure of imine methyltransferase. When the single nucleotide polymorphisms (SNP) loci of Rs1267369 and RS760537 in patients were homozygous alleles (AA type), the risk of vertebral fracture was significantly increased. Moreover, Mori et al. (2008) found that genetic variation of THSD7A and THSD4 was the decisive factor leading to osteoporosis in Japanese women. Two SNPs, rs1267369 and RS10851839, were found in THSD7A and THSD4 by genotyping the bone mineral density (BMD) of the patients’ femur and lumbar vertebra. When rs1267369 of the SNP in THSD7A was homozygous (AA), it was one of the risk factors of osteoporosis, while when rs10851839 of the SNP of THSD4 was homozygous (AA), it was one of the protective factors of osteoporosis. The mechanisms may be achieved by mediating TGF-β activity.

Studies have also proved that THSD7A is closely associated with membranous nephropathy (Tomas et al., 2014). Through serum analysis of patients with membranous nephropathy, the urinary protein level was positively correlated with the titer of THSD7A, and the serum THSD7A level was decreased with the activity degree in a course, which might affect the activity degree of the disease. Furthermore, animal studies suggested that the binding of a human THSD7A antibody with mouse THSD7A leads to proteinuria, which may be one of the pathogenesis of membranous nephropathy (Tomas et al., 2016).

Related gene chip studies have shown that THSD7A is highly expressed in the malignant tissues of prostate cancer, renal cell carcinoma, colorectal cancer, and breast cancer (Hoxha et al., 2016). THSD7A expression is closely related to the metastasis, differentiation, and invasion of related malignant tumors. Furthermore, Hou et al. (2017) found that Thsd7a gene knockout significantly reduced the proliferation, invasion, and migration of esophageal cancer cells and affected their differentiation and apoptotic rate. Furthur studies have found that THSD7A may impact the biological processes of cancer cells *via* multiple tumor-related pathways, such as mTOR, Wnt, and MAPK (Kuo et al., 2011). Moreover, some studies have indicated that THSD7A inhibits endothelial cell migration and angiogenesis by binding to αVβ3 integrin, a potential factor for targeted therapy for cancer and related vascular diseases (Wang et al., 2010).

Syndecan-4, an important member of the Syndecan family of transmembrane transporter polysaccharides, is a soluble heparan sulfate proteoglycan widely distributed in multi-system tissue cells showing significant protein changes in various disease states, such as inflammation, trauma, and tumor. It has a variety of physiological functions by binding with growth factors or cell-matrix. SDC4 has an important effect on intervertebral disc degeneration induced by inflammatory factors, such as TNF-α and IL-1β (Binch et al., 2016). Wang et al. (2011) found that SDC4 mRNA was significantly expressed in the degenerative intervertebral disc tissues, and the SDC4 expression was regulated by the pro-inflammatory factors TNF-α and IL-1β *via* the nuclear factor-kappa B (NF-κB) signaling pathway. SDC4 is closely associated with the activation of multiple growth factors and MMPs. In nucleus pulposus cells, inflammatory factors regulate the degradation of polyproteoglycan by affecting the expression of ADAMTS-4 and ADAMTS-5; moreover, the binding of ADAMTS-5 to SDC4 is a necessary step for the activation. This also suggests that the high-expression SDC4 induced by TNF-α and IL-1β is the main cause of polyproteoglycan degradation in human diseases. TNF-α and IL-1β alter the hydrophilicity and biomechanics of intervertebral discs, ultimately accelerating degeneration of the intervertebral disc. Wang et al. (2014) found that TNF-α and IL-1β induce MMP-3 expression in nucleus pulposus cells, in which SDC4 has an important role. TNF-α and IL-1β also activate the NF-κB pathway and regulate SDC4 expression, which may be the direction of targeted remission of the catabolic effects of MMP-3. Studies have indicated that MMPs, ADAMTS, and SDC4 are closely related to the process of intervertebral disc degeneration (Fujita et al., 2012; Godmann et al., 2015; Zou, 2015). Thus, SDC4 can accelerate EMC degradation and promote intervertebral disc degeneration by interacting with pro-inflammatory factors and matrix metalloproteinases.

CCL5, a CC subfamily chemokine, is located on human chromosome 17q11-32 and can be produced by various cells in humans (Herder et al., 2008). The main receptors of CCL5 include CCR1, 3, and 5 G protein-coupled receptors. As the most important receptor, CCL5 is the membrane protein CCR5, a surface marker of active Th1 cells (Liou et al., 2012). It has many effects, including the chemotactic effect on multiple types of cells, a strong activation effect on lymphocytes, regulatory effect on the growth and differentiation of cells. Recent studies have proved that CCL5 can promote the homing of stem cells and their migration to the damaged intervertebral disc cells. High expression of CCL5 mRNA in the degenerative intervertebral disc has also been proven (Kepler et al., 2013a), but its role in the process of the degenerative intervertebral disc remains unclear. Pattappa et al. (2014) suggested that chemokines stimulated the chemotactic movement of MSC to degenerative intervertebral disc tissue and induced tissue regeneration. They found that CCL5 has an important role in inducing MSC migration to degenerative intervertebral disc and increases the mRNA expression of CCL5-specific chemokine receptors in MSC. Thus, CCL5 is the main chemical attractant in MSC in degenerative intervertebral discs. Baek et al. (2011) and Ponte et al. (2007) also reported that MSCs migrated to CCL5 and other designated chemokines. Besides, related factors endow CCL5 with a greater chemotactic migration effect on MSC and improve the therapeutic effect of MSC therapy. Due to degeneration, intervertebral disc cells secrete CCL5 to induce the migration of MSCs to degenerative tissues. The main factor promoting the migration might be related to the high expression of receptors promoting chemotaxis on the cell surface (Honczarenko et al., 2006), which is greatly important to the regeneration and repair of damaged tissues in the early stage of intervertebral disc degeneration. However, the relevant mechanism needs further verification.

Aquaporins (AQPs) are a family of membrane channel proteins with a tetramer structure related to transmembrane water transport discovered in 1988. Scientists have gradually realized that water could enter cells through transport channels except for simple diffusion and infiltration. The intervertebral disc is a tissue with high water content, which is as high as 80% in the nucleus pulposus in childhood and 70% in older age. The common feature of intervertebral disc degeneration is reduced water content, so the role of water in maintaining its normal physiological function is important. AQP3 has an important role in the intervertebral disc tissue, acting as a water transport channel for intervertebral disc cells. AQPs are expressed in the intervertebral disc tissue. Richardson et al. (2008) found that AQP3 was expressed in human intervertebral disc cells, with the highest expression in the nucleus pulposus. Furthermore, Cao et al. (2011) proved that the AQP3 expression was negatively correlated with the degree of intervertebral disc degeneration. It has been suggested that AQP3 might participate in intervertebral disc degeneration, and the low expression might be one of the reasons for the degeneration. In addition, abnormal AQP3 expression-induced water absorption disorder also affects energy synthesis and metabolism of intervertebral disc cells (Zhang et al., 2012). AQP3 provides water and glycerin for nucleus pulposus cells to complete their anaerobic glycolysis. At the same time, cell metabolites are discharged to improve the external environment of cells, having an important role in maintaining a moderately acidic environment of cells. There is no direct blood supply for the intervertebral disc, so the nutritional pathway is very important for maintaining normal physiological functions of the intervertebral disc (Yao. et al., 2014). AQP3 may participate in intervertebral disc degeneration *via* the NF-κB signaling pathway. In addition, Yao. et al. (2014) reported that the NF-κB signaling pathway could activate a large amount of pro-inflammatory factors that inhibit AQP3 expression. The NF-κB signaling pathway activates apoptosis, which may decrease AQP3 expression. Intervertebral disc degeneration and dehydration decrease the AQP3 expression, which in turn results in the obstruction of the intervertebral disc nutrient supply, causing changes in the cellular acid/base environment, as well as the inflammatory response of the intervertebral disc and activation of NF-κB expression.

The study showed that the expression of AQP3 and CCL5 in the ruptured intervertebral disc was significantly up-regulated, while the expression of SDC4 was down-regulated; this reduced the inflammatory response of herniated nucleus pulposus, slowed down the degradation of the extracellular matrix, and contributed to the recovery of the dynamic balance of the extracellular matrix. Up-regulation of AQP3 could help restore the normal physiological function of the intervertebral disc, the energy synthesis, and metabolism of intervertebral disc cells, which is important for the nutritional supply of the intervertebral disc. Both SDC4 and AQP3 were closely associated with inflammatory factors and related inflammatory pathways. SDC4 could form positive feedback with inflammatory factors and mediate intervertebral disc degeneration through inflammatory pathways together. Moreover, inflammatory factors could inhibit AQP3 expression and affect the nutritional supply of the intervertebral discs. The down-regulation of SDC4 and up-regulation of AQP3 occurred simultaneously, indicating the presence of self-repair of ruptured herniated tissue. The up-regulation of CCL5 facilitated the migration of MSCs and repair of the damaged disc tissue. Cell migration, cell adhesion, matrix steady-state in multicellular organisms, as well as ECM-receptor interaction, vasopressin-regulated water reabsorption, chemokine signaling pathways, amino acid synthesis, and metabolism further demonstrated the self-repair ability of the damaged tissue in the intervertebral disc.

In conclusion, we found that gene expression changes after ruptured disc tissue herniates through the posterior longitudinal ligament. Our data suggest that THSD7A can be used as a characteristic differentially expressed gene of the ruptured nucleus pulposus. Based on the understanding of differentially expressed genes, including CCL5, AQP3, and SDC4, combined with the related biological processes and pathways analyzed by bioinformatics, the up-regulation of AQP3 and CCL5 and down-regulation of SDC4 were preliminarily predicted to alleviate the lack of cell nutrient supply, reduce the degradation rate of the external matrix, repair the damaged disc tissue, and slow the progression of disc degeneration.

## Conclusion

THSD7A can be used as the characteristic differentially expressed gene of human ruptured nucleus pulposus. CCL5, AQP3, and SDC4 were preliminarily predicted to improve self-repair of the damaged intervertebral disc tissue repair chemotaxis of stem cell migration, increasing water absorption of nucleus pulposus cells, and inhibiting the inflammatory response, thus delaying the process of intervertebral disc degeneration.

.

## Data Availability

The original contributions presented in the study are included in the article/supplementary material, further inquiries can be directed to the corresponding author.
